# tRNA-Derived Small RNAs and Their Potential Roles in Cardiac Hypertrophy

**DOI:** 10.3389/fphar.2020.572941

**Published:** 2020-09-17

**Authors:** Jun Cao, Douglas B. Cowan, Da-Zhi Wang

**Affiliations:** Department of Cardiology, Boston Children’s Hospital, Harvard Medical School, Boston, MA, United States

**Keywords:** tRNA-derived small RNAs (tsRNAs), tRNA halves, tRNA fragments, heart, cardiac hypertrophy, mitochondria, non-coding RNAs, cardiovascular disease

## Abstract

Transfer RNAs (tRNAs) are abundantly expressed, small non-coding RNAs that have long been recognized as essential components of the protein translation machinery. The tRNA-derived small RNAs (tsRNAs), including tRNA halves (tiRNAs), and tRNA fragments (tRFs), were unexpectedly discovered and have been implicated in a variety of important biological functions such as cell proliferation, cell differentiation, and apoptosis. Mechanistically, tsRNAs regulate mRNA destabilization and translation, as well as retro-element reverse transcriptional and post-transcriptional processes. Emerging evidence has shown that tsRNAs are expressed in the heart, and their expression can be induced by pathological stress, such as hypertrophy. Interestingly, cardiac pathophysiological conditions, such as oxidative stress, aging, and metabolic disorders can be viewed as inducers of tsRNA biogenesis, which further highlights the potential involvement of tsRNAs in these conditions. There is increasing enthusiasm for investigating the molecular and biological functions of tsRNAs in the heart and their role in cardiovascular disease. It is anticipated that this new class of small non-coding RNAs will offer new perspectives in understanding disease mechanisms and may provide new therapeutic targets to treat cardiovascular disease.

## Introduction

Small noncoding RNA (sncRNA) usually refers to RNA molecules less than 200 nucleotides (nt) in length, which are transcribed from DNA, but not translated into protein. SncRNAs include but are not limited to microRNAs (miRNAs), endogenous short interfering RNAs (siRNAs), small nuclear RNAs (snRNAs), small nucleolar RNAs (snoRNAs), piwi interacting RNAs (piRNAs), ribosomal RNA derived fragments (rRFs), transfer RNAs (tRNAs), and their derived small RNAs (tsRNAs) (Mattick and Makunin, 2006; Kirchner and Ignatova, 2015; Wei et al., 2017; Lambert et al., 2019). With the advance of high-throughput RNA sequencing (Giraldez et al., 2018; Liu et al., 2019), new classes of sncRNAs are being discovered and studied.

Different sncRNAs exert diverse but specific functions in cells. For example, miRNAs and siRNAs regulate gene expression by tuning mRNA stability and translational efficiency (Valencia-Sanchez et al., 2006). In addition, snRNAs promote proteome diversity by regulating pre-mRNA splicing (Valadkhan, 2005), snoRNAs modify rRNAs, snRNAs, and even mRNAs with 2′-O-methylated nucleotides (Kiss, 2002), and piRNAs contribute to transposon silencing (Ozata et al., 2019). Studies have also uncovered multiple molecular pathways and functions related to a single type of sncRNAs (Kiss, 2002; Pillai, 2005; Rojas-Rios and Simonelig, 2018). Consequently, it is conceivable that new mechanisms and functions of sncRNAs remain to be discovered.

The important and various molecular functions of sncRNAs in cells make them vital regulators in both physiological and pathological conditions, such as during development (Mendell, 2008; Chen and Wang, 2012; Rojas-Rios and Simonelig, 2018), cancer progression (Ling et al., 2013; Peng and Croce, 2016; Romano et al., 2017), neurodegenerative disease (Rege et al., 2013; Watson et al., 2019), and cardiovascular disease (Romaine et al., 2015; Zhou et al., 2018). Among sncRNAs, tRNA-derived small RNAs (tsRNAs) have gained considerable attention as these molecules have various subtypes that are generated by different mechanisms and exert a variety of critical functions in cells. Moreover, they are also implicated in development and disease. As tsRNAs are expressed in the heart and participate in the function of this organ, we will focus on their biogenesis and function, and we will discuss potential research opportunities to study the role of tsRNAs in the heart.

## Biogenesis and Expression of Nuclear and Mitochondrial Encoded Transfer RNAs

In eukaryotic cells, both the nucleus and mitochondria encode tRNA genes, producing two types of tRNAs—cytoplasmic tRNAs and mitochondrial tRNAs (mt-tRNAs). There are more than 500 tRNA genes either identified or predicted to exist in humans (Chan and Lowe, 2009; Chan and Lowe, 2016). About half of them are verified to be actively expressed genes (Schimmel, 2018), which are transcribed to 51 isoacceptor tRNA types and decode to 61 codons for translation (Mahlab et al., 2012; Chan and Lowe, 2016). Therefore, some codons are derived from multiple tRNA genes in the human genome. In contrast, mt-tRNAs are transcribed from only 22 mt-tRNA genes in the mitochondrial genome. These mt-tRNA genes play critical roles in assisting translation of mitochondrial proteins with less redundancy. Mutations in mt-tRNAs have also been implicated as important components in cardiovascular diseases such as coronary heart disease (Jia et al., 2013; Jia et al., 2019), cardiomyopathy (Taniike et al., 1992; Casali et al., 1995; Arbustini et al., 1998; Marin-Garcia et al., 2001; Hsu et al., 2008), and hypertension (Liu et al., 2009; Wang et al., 2011; Jiang et al., 2016). It is worth noting that cytoplasmic tRNAs can be imported to mitochondria, which suggests that they may also play essential roles in mitochondrial biology and disease (Rubio et al., 2008; Mercer et al., 2011).

**Figure 1:**
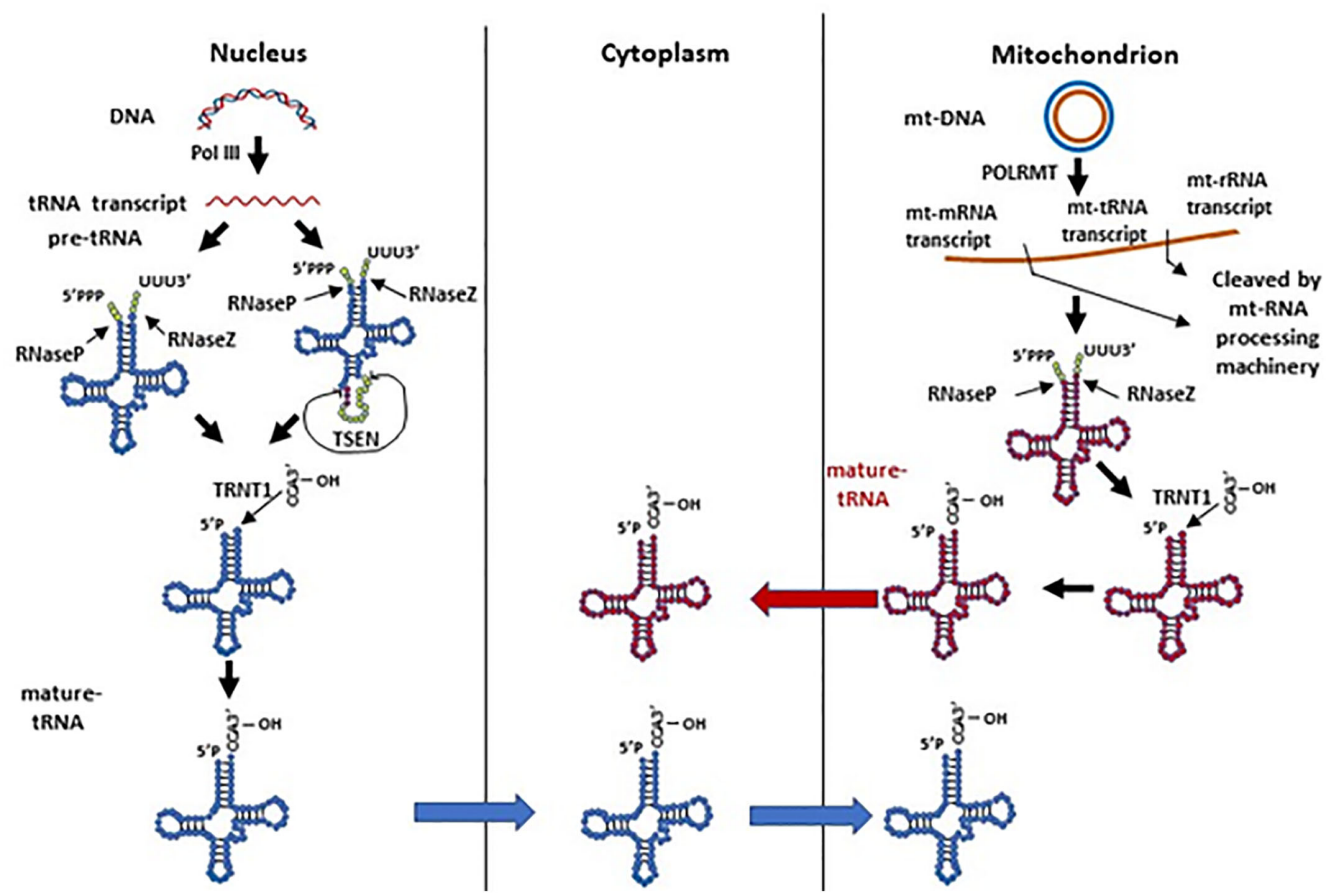
The biogenesis of human nuclear and mitochondrial encoded tRNAs. Pre-tRNAs can be transcribed by Pol III in the nucleus or by POLRMT in mitochondria. The pre-tRNAs have 5’-leader and 3’-trailer sequences, which are trimmed by RNase P and RNase Z, respectively. A minority of nuclear pre-tRNAs have introns, which are spliced by the TSEN complex. A single “CCA” sequence is then added to all trailer trimmed 3’-ends of human tRNAs by the TRNT1 protein. Processed nuclear tRNAs are transported to the cytoplasm, while mitochondrial tRNAs predominantly remain in mitochondria.

Human nuclear tRNA genes are initially transcribed by RNA polymerase III (RNA Pol III) as pre-tRNAs, which contain 5’-leader and 3’-trailer sequences (Phizicky and Hopper, 2010). The 5’-leader and 3’-trailer are trimmed by RNase P (Frank and Pace, 1998) and RNase Z (Maraia and Lamichhane, 2011), respectively (**Figure 1**). Following that, a single “CCA” sequence is added to all trailer trimmed 3’-ends of human tRNAs by tRNA nucleotidyl transferase (TRNT1) (Anderson and Ivanov, 2014). A minority of human pre-tRNAs have intron sequences, which are spliced by a nuclear tRNA splicing endonuclease (TSEN) during tRNA processing (Anderson and Ivanov, 2014). TSEN cleaves a pre-tRNA containing intron into three parts: a 5’-exon with a 2’-3’-cyclic phosphate at its 3’ end, a 3’-exon with a 5’-hydroxyl group (5’-OH) at its 5’-end, and the excised intron (Anderson and Ivanov, 2014). After the cleavage, the 5’-exon and 3’-exon are ligated to become a mature tRNA before being transported to the cytoplasm. In contrast, mitochondrial tRNA genes are transcribed by mitochondrial RNA polymerase (POLRMT) along with mitochondrial rRNA and mRNA genes in a long mitochondrial polycistronic DNA template (Suzuki et al., 2011). The mt-tRNA transcripts are then cleaved from rRNA and mRNA transcripts by mitochondrial RNA-processing enzymes according to the mt-tRNA “punctuation” model (Ojala et al., 1981; Rossmanith et al., 1995; Barchiesi and Vascotto, 2019). Similar to cytoplasmic pre-tRNAs, mitochondrial pre-tRNAs require RNase P and ELAC2 (mitochondrial RNase Z) to remove the 5’-leader and 3’-trailer, respectively (Rossmanith et al., 1995; Brzezniak et al., 2011; Suzuki et al., 2011; Haack et al., 2013; Siira et al., 2018). Finally, a “CCA” sequence is attached to the 3’ terminus of mt-tRNA by a mitochondrial TRNT1 to complete mt-tRNA maturation (Suzuki et al., 2011). Mature human cytoplasmic tRNAs are usually 76 to 93 nts in size and form a cloverleaf-like secondary structure with stem and loop regions, and they are eventually compacted into an L-shape tertiary structure (Schimmel, 2018). Mature mt-tRNAs range from 59 to 75 nts in size with smaller stem and loop regions, and some of them lack entire domains (Schimmel, 2018). The mt-tRNAs form a non-canonical cloverleaf-like secondary structure (Helm et al., 1998; Suzuki et al., 2011; Schimmel, 2018) and L-shape tertiary structure (Messmer et al., 2009; Salinas-Giege et al., 2015). It is worth noting that mitochondrial tRNA-lookalikes have been detected in the nuclear genome in human and some other primates (Telonis et al., 2014; Telonis et al., 2015a), suggesting that mitochondria may not be the sole source of mitochondrial tRNAs. However, it remains elusive 1) whether these nuclear-encoded mitochondrial tRNA lookalikes are actively expressed; 2) if so, whether these tRNAs are transported to cytoplasm and/or mitochondria; and 3) what functions they may exert in different cellular compartments.

Although it seems that tRNA processing undergoes two parallel and separate systems in the nucleus and mitochondrion, we should not rule out the possibility of tRNA dynamics between these organelles (**Figure 1**). As mentioned above, nuclear-encoded tRNAs are able to shuttle between the cytoplasm and mitochondria (Rubio et al., 2008; Mercer et al., 2011); mitochondrial tRNA lookalikes exist in the nuclear genome (Telonis et al., 2014; Telonis et al., 2015a). On the other hand, though mature human mt-tRNAs mainly function in the mitochondria for mitochondrial gene translation, they have also been reported to be in the cytoplasm (Maniataki and Mourelatos, 2005). Moreover, the tRNA splicing endonuclease TSEN, which is expressed solely in the nucleus in humans, is located on the mitochondrial surface in yeast (Dhungel and Hopper, 2012; Hopper and Nostramo, 2019). In yeast, nuclear pre-tRNAs with introns have to be exported to the cytoplasm and spliced on the surface of mitochondria (Dhungel and Hopper, 2012; Hopper and Nostramo, 2019). These spliced tRNAs are subsequently modified in the cytoplasm, and imported back to the nucleus for additional modification before being again exported to the cytoplasm to carry out their intended function (Dhungel and Hopper, 2012; Hopper and Nostramo, 2019). Even though human cytoplasmic tRNA processing does not have this splicing step on mitochondria like yeast, it remains unclear whether these organelles play any other roles in cytoplasmic tRNA processing or modification, and whether mt-tRNAs have any function in the cytoplasm.

## Transfer RNA-Derived Small RNAs (tsRNAs)

**Figure 2:**
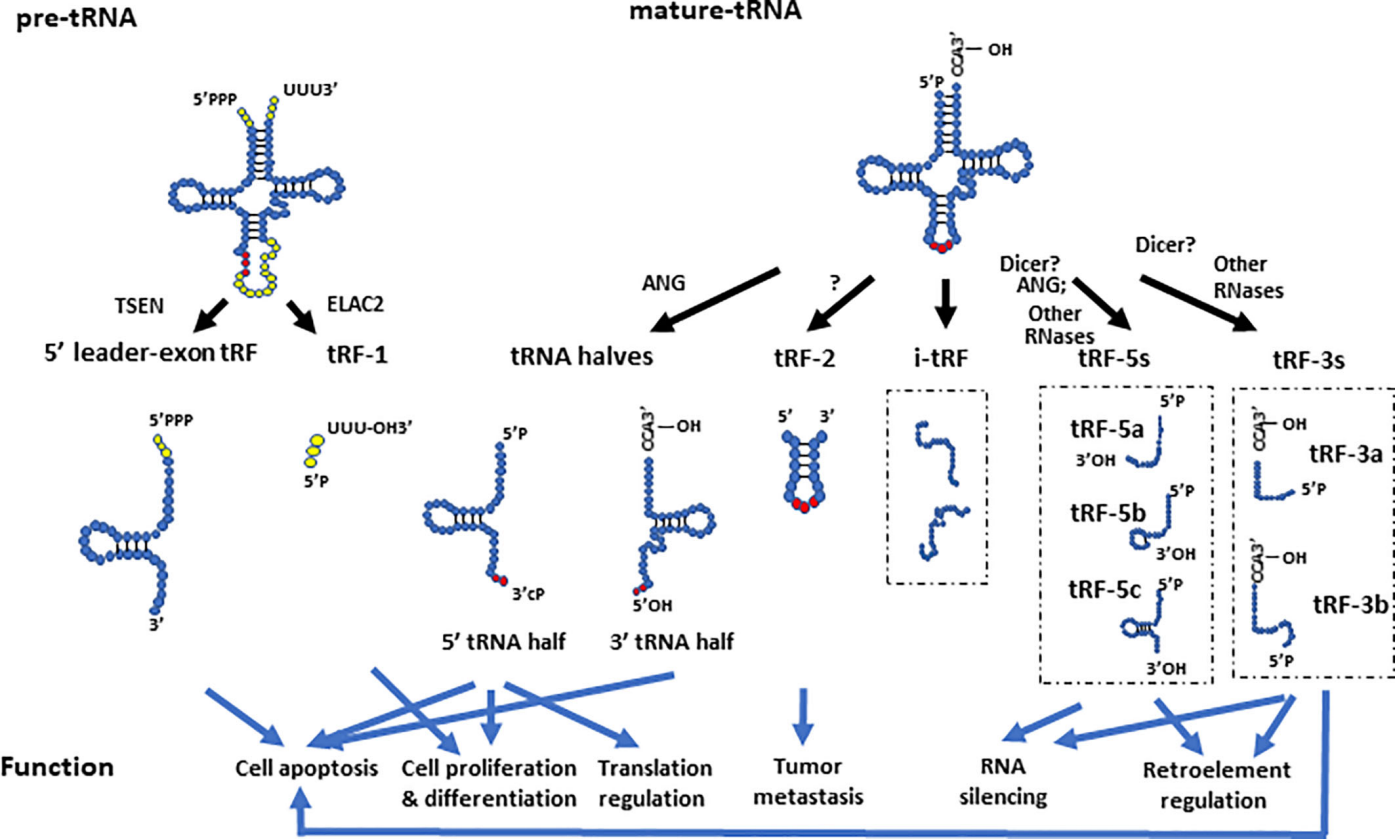
The biogenesis and function of different tsRNAs derived from pre- and mature- tRNAs. The 5’ leader-exon tRFs and tRF-1s are generated from cleavage of pre-tRNAs by TSEN and ELAC2, respectively. The 5’- and 3’- tRNA halves are generated by cleavage of mature tRNAs at the anticodon region by ANG. tRF-2s contain anticodon stem and loop regions of mature tRNAs. The tRF-5 group includes tRF-5a, tRF-5b, and tRF-5c, which are generated by endonucleolytic cleavage of mature tRNAs at D loop, D stem, and the stem regions between the D stem and anticodon loop, respectively. The tRF-3 group includes the tRF-3a and tRF-3b subgroups, which are generated by endonucleolytic cleavage of mature tRNAs at different locations of their T arms. The i-tRFs are generated from internal parts of tRNAs, whose 5’ termini start from the second or subsequent nucleotide of mature tRNAs. They are usually about 36 nts in size, and have various subtypes. This figure only showed two examples of i-tRFs. The different tsRNAs contribute to a variety of molecular processes such as translational regulation, RNA silencing, and retro-element regulation. They are also involved in tumor metastasis, apoptosis, cell proliferation, and differentiation.

In general, tsRNAs can be grouped into two categories based on their size and biogenesis: tRNA halves (or tRNA-derived, stress-induced RNAs, also known as tiRNAs) and tRNA-derived fragments (also known as tRFs) (Anderson and Ivanov, 2014) (**Figure 2**). The tRNA halves or tiRNAs refer to the tsRNAs that are half the size of tRNAs. The tRFs usually refer to even smaller tsRNAs, which have a range of sizes based on their cleavage.

It is worth noting that “tRFs”, “tRNA halves”, and “tsRNAs” were sometimes used interchangeably because the nomenclature for them was not initially clear. For instance, some studies referred to “tRNA halves” as “tRFs” (Ivanov et al., 2011; Li et al., 2016; Sharma et al., 2016), while other studies referred to “tsRNAs” as “tRFs” (Anderson and Ivanov, 2014; Liapi et al., 2020). Therefore, we advise authors to scrutinize the literature carefully when reading and/or citing them so as to obtain extensive and precise information for each category of tsRNAs.

### Transfer RNA Halves (tiRNAs)

The tRNA halves are generated by specific cleavage in or close to the anticodon region, which leads to 30-50 nt long 5’ and 3’ tRNA halves (Anderson and Ivanov, 2014). A number of studies showed that tRNA halves are expressed minimally in human cell lines, but are induced during stress conditions including oxidative stress (Thompson et al., 2008; Yamasaki et al., 2009), nutritional deficiency (Fu et al., 2009), hypoxia (Fu et al., 2009), heat shock (Fu et al., 2009; Yamasaki et al., 2009), UV irradiation (Yamasaki et al., 2009), and viral infection (Wang et al., 2013). Since tRNA halves are part of mature tRNAs, there is a question whether these tRNA halves are artificial degradation by-products derived from tRNAs that have real functions in cells and tissues. Multiple pieces of evidence have demonstrated the specificity of tiRNA biogenesis, implying that they may actually possess unique functions in the cell. First, several studies have shown the level of tRNA halves do not always correlate with the levels of their cognate mature tRNAs (Gebetsberger and Polacek, 2013; Honda et al., 2015; Krishna et al., 2019). For instance, arsenite stress induced Met-tRNA halves without affecting their parental mature Met-tRNA levels (Yamasaki et al., 2009). Second, tRNA halves were enriched in fetal mouse liver, while expressed at low levels in the heart (Fu et al., 2009), which suggests that tRNA halves have specific expression patterns in different tissues. In addition, Gly-, Val-, Met-, and Arg- tRNA halves were dramatically increased upon nutritional starvation, while Tyr-tRNA halves were not induced (Fu et al., 2009); thereby, reinforcing the idea of specific biogenesis of tRNA halves in different conditions. In line with this work, a recent study identified tRNA halves in mouse serum by RNA-Seq, and revealed that Gly- and Val- tRNA halves together account for ~90% of circulating tRNA halves, whereas the majority of these molecules are below detectable limits (Dhahbi et al., 2013). Interestingly, 5’ tRNA halves were found to be much more enriched than 3’ tRNA halves in serum (Dhahbi et al., 2013), which indicates a specific role for 5’ tRNA halves compared with 3’ tRNA halves in mouse serum (Dhahbi et al., 2013). However, we would like to point out that we could not rule out the possibility that 3’ tRNA halves may be underestimated. 3’-tRNA halves were found to be charged with amino acids at their 3’-end in cancer cells, which may prevent their detection in small RNA sequencing or PCR amplification based methods involving adaptor ligations (Honda et al., 2015). In addition, some of the 3’-tRNA halves, such as 3’-His-tRNA halves, cannot be detected by RACE due to the presence of guanine at their 5’ end, as that residue is often modified to 1-methyl-guanosine (m^1^G), which inhibits reverse-transcription (Honda et al., 2015). In summary, the expression levels of tRNA halves vary across different conditions, tissues, species, and 5’ vs 3’ origins, and they do not always correlate with their cognate mature tRNA expression levels, which together demonstrate a specificity in their biogenesis. Although individual tRNA halves have been characterized in different cells or tissues, how genome wide tRNA halves are expressed in various tissues and conditions remains an open question.

tRNA halves are recognized to be generated by angiogenin cleavage during stress (Fu et al., 2009; Yamasaki et al., 2009). Angiogenin (ANG), also known as ribonuclease 5, is a secreted ribonuclease that cleaves tRNAs into tRNA halves both *in vitro* and *in vivo* (Fu et al., 2009; Yamasaki et al., 2009; Su et al., 2019). Exogenous expression of ANG (Fu et al., 2009) or the knockdown of an ANG inhibitor (RNH1) (Yamasaki et al., 2009) promotes the generation of tRNA halves, while knock down of ANG itself reduces the levels of stress-induced tRNA halves (Yamasaki et al., 2009). After ANG cleaves tRNAs at the anticodon region, it leaves 2’- 3’- cyclic phosphates at the 3’ ends and hydroxyl groups at 5’ ends of tRNA halves (Yamasaki et al., 2009). It is worth noting that since these 2’-3’-cyclic phosphates may inhibit detection by small RNA sequencing or PCR amplification based methods involving adaptor ligations, there might be an underestimation of existing tRNA halves when performing the above quantification methods. The 2’- 3’- cyclic phosphates at the 3’ ends and hydroxyl groups at 5’ ends of tRNA halves differentiate tRNA halves from other small RNAs cleaved by Dicer or RNase III type enzymes, which usually have a 5’ phosphate rather than a 5’ hydroxyl group (Kumar et al., 2016). Although ANG is a major contributor for tRNA halves, it is not clear what other nucleases may also contribute to the generation of tRNA halves. Additional research would provide a greater understanding of the biogenesis of tRNA halves.

### Transfer RNA-Derived Fragments (tRFs)

tRFs are even smaller fragments derived from mature tRNAs or pre-tRNAs—usually 14–32 nt in length. Similar to tRNA halves, various tRFs have distinct expression patterns in different tissues (Kawaji et al., 2008), and there is no correlation between parental tRNA levels and their derived tRF levels (Kim et al., 2017). In addition, certain parental tRNAs only produce certain subtypes of tRFs (Su et al., 2019). Even when derived from the same parental tRNAs, some tRFs are differentially expressed (Li et al., 2012; Kumar et al., 2014; Telonis et al., 2015b), which is referred to as asymmetric processing of tRFs from mature tRNAs (Li et al., 2012). For instance, a recent research study examined the abundance of tRFs in transcriptomic data from 452 healthy people and 311 breast cancer patients, and found that different tRFs form the same parental tRNAs do not have correlated abundance (Telonis et al., 2015b). In addition, it identified specific genomic loci clusters that may be responsible for generation of particular types of tRFs (Telonis et al., 2015b), which suggests that the abundance of particular tRFs, at least partially, depends on their genomic location. Collectively, these studies indicate that tRFs are likely not random degradation by-products of tRNAs, but seem to have their own specific biogenesis and function independent of their parental tRNAs in different biological conditions.

Depending on their cleavage sites and origin, tRFs can be divided into several groups such as tRF-1s (also known as 3’U tRFs), tRF-2s, tRF-3s (also known as 3’CCA tRFs), tRF-5s, i-tRFs (Telonis et al., 2015b), and 5’ leader-exon tRFs (Gebetsberger and Polacek, 2013; Goodarzi et al., 2015; Shen Y. et al., 2018). The tRF-2s, tRF-3s, tRF-5s, and i-tRFs are derived from mature tRNAs, whereas tRF-1s and 5’ leader-exon tRFs are generated from pre-tRNAs (Shen Y. et al., 2018). As discussed above, a mature tRNA forms a cloverleaf secondary structure. Cloverleaf-like tRNAs have four arms, which are designated as the acceptor stem, dihydrouridine (D) stem-loop (D arm), anticodon stem-loop, and TψC stem-loop (T arm) (Schimmel, 2018). tRF-2s are a newly discovered type of tRF identified in breast cancer cells, and they primarily contain anticodon stem and loop regions of tRNAs (Goodarzi et al., 2015; Kumar et al., 2016). This type of tRF is stress sensitive, and is significantly increased under hypoxic conditions (Goodarzi et al., 2015). The tRF-3s and tRF-5s are generated by endonucleolytic cleavage of mature tRNAs at the T arm and D arm, respectively (Anderson and Ivanov, 2014). A recent study sequenced tRFs in HEK293 cells and divided tRF-5s to three subtypes: tRF-5a (~15 nt in size), tRF-5b (~22 nt in size) and tRF-5c (~32 nt in size), which are generated by endonucleolytic cleavage of mature tRNAs at D loop, D stem, and the stem region between the D stem and anticodon loop, respectively (Kumar et al., 2014). The tRF-5cs may straddle the categories of tRF-5s and tRNA halves, as it is known that ANG can cleave tRNAs before the anticodon region which could potentially generate tRNA halves shorter than the typical 35 nts (Honda et al., 2015; Shigematsu and Kirino, 2017). The tRF-3s were classified into two sub-classes based on their tRF sequencing in HEK293 cells: tRF-3a (~18 nt in size) and tRF-3b (~22 nts in size), which are generated by endonucleolytic cleavage of mature tRNAs at different areas of their T arms (Kumar et al., 2014). It is worth noting that the subgroups of tRFs are not strictly defined by length, as tRF size may vary in different tissues or biological conditions. For example, additional tRF-5s with lengths of 20, 26, 33, and 36 nts as well as tRF-3s with lengths of 33 and 36 nts, were identified in a dataset from lymphoblastoid cell lines (Telonis et al., 2015b). The i-tRFs (internal fragments) were newly identified tRFs in breast cancer samples and breast cancer cell lines (Telonis et al., 2015b). This type of tRFs correspond to an internal part of mature tRNAs, which means that they neither start from the exact 5’ terminus (the first nucleotide of 5’ terminus) nor end at the 3’ terminus (any base in 3’ terminal “CCA” sequence) of mature tRNAs (Telonis et al., 2015b). Instead, the 5’ terminus of i-tRFs starts from the second or subsequent nucleotide of mature tRNAs, and they are usually about 36 nts in size (Telonis et al., 2015b). This differentiates this type of tRFs from tRF-3s and tRF-5s.

Aside from tRNA halves, small tRFs usually possess both a 5’-phosphate and 3’-hydroxyl group (Haussecker et al., 2010). Since Dicer recognizes the 5’-phosphate group of small RNAs (Park et al., 2011), it was initially considered to be required for the processing of tRF-3s and tRF-5s. However, there are studies that indicate the existence of both Dicer-dependent and Dicer-independent biogenesis of tRFs. Therefore, it remains unknown if Dicer is indispensable for generation of tRFs or whether it is required for some types of tRFs under certain conditions. Some tRF-3s were detected by high-throughput sequencing in wild-type mouse embryonic stem cells (mESCs), but not in mESCs with a Dicer 1 deletion, which suggests that the generation of tRF-3s requires Dicer 1 (Babiarz et al., 2008). In line with this work, depletion of Dicer significantly reduced the abundance of tRF-5s derived from Gln-tRNAs in HeLa cells (Cole et al., 2009), which reinforces the importance of Dicer for the generation of tRFs. On the other hand, there are a number of studies that show Dicer is dispensable for tRF biogenesis. For instance, knockout of Dicer or DGCR8 (a microprocessor complex subunit) did not exert any effect on tsRNA expression in mESCs (Li et al., 2012). Consistently, mutation of DICER1/DGCR8 did not decrease tRF expression in mouse ESCs (Kumar et al., 2014). In addition, ANG, which has been identified as an endonuclease contributing to the biogenesis of tRNA halves, was found to contribute to the generation of tRFs (Li et al., 2012). RNase A, RNase I, and RNase T1 were also found to be able to cleave tRNAs to tRFs, and the tRFs derived from RNase T1 cleavage were different from the ones digested by ANG and RNase A/I (Li et al., 2012).

tRF-1s derived from the 3’ end of pre-tRNAs contain a stretch of U residues that are usually produced by RNA polymerase III (Lee et al., 2009). Since the tRF-1s are generated from pre-tRNAs, they would be assumed to reside in the nucleus; however, tRF-1s can also be located in the cytoplasm (Lee et al., 2009; Kumar et al., 2014). The tRF-1s were found to be cleaved by ELAC2 in the cytoplasm, and tRF-1 expression levels are regulated by ELAC2 in prostate cancer cell lines (Lee et al., 2009). On the other hand, Dicer was found not to be a regulator for tRF-1 in HEK293 cells (Haussecker et al., 2010). Another type of tRFs derived from pre-tRNAs are the 5’ leader-exon tRFs, which were discovered in mouse embryonic fibroblasts (MEFs) with RNA sequencing (Hanada et al., 2013). This type of tRFs contain a complete 5’ leader triphosphate group followed by the 5’ exon tRNA sequence, and their expression decreases upon TSEN depletion (**Figure 2**) (Hanada et al., 2013). The 5’ leader-exon tRFs are stress sensitive, as they were induced by H_2_O_2_, but not ANG in MEFs (Hanada et al., 2013).

## Molecular and Biological Functions of tsRNAs

The tRNA halves and different tRFs have specific molecular functions that allow them to play distinct roles in different conditions. The tsRNAs regulate a variety of biological processes including translation (Emara et al., 2010; Ivanov et al., 2011) (Kim et al., 2017), RNA stability (Haussecker et al., 2010; Kumar et al., 2014; Kuscu et al., 2018), retro-element reverse transcription and post-transcription (Schorn et al., 2017; Boskovic et al., 2020), apoptosis (Saikia et al., 2014), cell proliferation and differentiation (Honda et al., 2015; Krishna et al., 2019). These characteristics involve them in many physiological and pathological conditions, including development (Krishna et al., 2019), aging (Dhahbi et al., 2013), neurodegenerative diseases (Hanada et al., 2013), cancer (Honda et al., 2015), and cardiovascular diseases (Shen L. et al., 2018) (**Table 1**) (**Figure 2**).

**Table 1:** Summary of transfer RNA-derived small RNAs (tsRNAs) and their biological functions.

Tissue/Cell line	Inducers	tsRNA type	Examples of tsRNAs	Biological functions	Molecular mechanisms	Reference
U2OS	ANG	5’-tRNA halves	5’-Ala-tRNA halves; 5’-Cys-tRNA halves	Repress translation	The 5’-tRNA halves cooperate with YB-1 to displace eIF4G/A from uncapped and capped mRNAs, thus inhibit translation.	(Ivanov et al., 2011)
Mouse Embryonic Fibroblasts	ANG	5’-tRNA halves; 3’-tRNA halves	5’-Asp-tRNA halves; 3’-Arg-tRNA halves; 3’-Gly-tRNA halves; 3’-Ala-tRNA halves	Inhibit cell apoptosis	The tRNA halves bind to Cyt c and prevent Cyt c from binding to Apaf-1 and activating caspase pathways.	(Saikia et al., 2014)
Breast cancer and prostate cancer cells	ANG and sex hormone	5’-tRNA halves	5’-Asp-tRNA halves; 5’-His-tRNA halves	Promote cell proliferation	Unknown	(Honda et al., 2015)
Mouse embryonic stem cells	Endogenously detected	5’-tRNA halves	5’-Gln-tRNA halves; 5’-Gly- tRNA halves; 5’-Glu- tRNA halves; 5’-Val- tRNA halves	Facilitate cell differentiation	The 5’- tRNA halves interact with IGF2BP1, and prevent IGF2BP1 from binding to and stabilizing the transcripts of c*-myc*, which is a pluripotency-promoting factor.	(Krishna et al., 2019)
Mouse sperms	High-fat diet	5’-tRNA halves	5’-Gly-tRNA halves; 5’-Glu- tRNA halves; 5’-Val- tRNA halves	Promote intergenerational inheritance of metabolic disorder	May affect expression of genes involving apoptosis, autophagy, oxidative stress, glucose input etc.	(Chen et al., 2016)
Breast cancer cells	Endogenously detected	tRF-2s	tRF-Gly; tRF-Asp; tRF-Glu; tRF-Tyr	Suppress cancer progression and metastasis	The tRF-2s displace 3’UTR of oncogenic transcripts from protein YBX1, which reduces stability of oncogenic transcripts.	(Goodarzi et al., 2015)
Hela, HCT-116 cells	Endogenously detected	tRF-3s	tRF-Leu	Promote cell viability	The tRF-3s interact with ribosomal protein mRNAs *RPS28* and *RPS15* to enhance their translation, which results in fine-tuning of gene expression in cells.	(Kim et al., 2017)
HEK293T	Endogenously detected	tRF-3s	tRF-Leu; tRF-Cys	RNA silencing	The tRFs target RNAs by base pairing, and associate Argonaute-GW182 containing RISC to mediate gene silencing.	(Kuscu et al., 2018)
Mouse stem cells	Endogenously detected	tRF-3s	tRF-Lys	Inhibit retrotransposition	22nt tRF-3s mediate post-transcriptional gene silencing; 18nt tRF-3s inhibit reverse transcription of retrotransposons.	(Schorn et al., 2017)
Human and mouse embryonic stem cells	Endogenously detected	tRF-5s	tRF-Gly	Inhibit retroelement transcription; regulate Cajal body biogenesis	The tRFs positively regulate histone genes, which repress retroelement transcription; the RNA binding proteins hnRNPF and hnRNPH bind to the tRFs, which are required for generation of Cajal body.	(Boskovic et al., 2020)
HEK293; A549; MCF7, mouse tissue etc.	Endogenously detected	tRF-5s	tRF-Tyr; tRF-Asp; tRF-Lys; tRF-Gly; tRF-Arg etc.	May regulate RNA silencing	The tRF-5s are associated with Argonaute proteins.	(Kumar et al., 2014)
Breast cancer samples and breast cancer cell lines	Endogenously detected	i-tRFs	tRF-Asp, tRF-Gly, mitochondrial tRF-Glu	unknown	unknown	(Telonis et al., 2015b)
HEK293, HCT-116 cells	Endogenously detected	tRF-1s	tRF-Ser	The antisense sequence of the tRFs enhances RNA silencing	The antisense sequence of tRFs enhances Ago2 loading to duplexed double-stranded RNA.	(Haussecker et al., 2010)
Prostate cancer cell lines	Endogenously detected	tRF-1s	tRF-Ser	Promote cell proliferation	Unknown	(Lee et al., 2009)
Mouse embryonic stem cells; spinal cord	CLP1 depletion; H_2_O_2_	5’ leader-exon tRFs	tRF-Tyr	Promote motor neuron loss	The tRFs may contribute to neuron loss in CLP1 knockout mice by coupling to p53 dependent cell death.	(Hanada et al., 2013)

### tRNA Halves (tiRNAs)

The most well-characterized function of tRNA halves is their inhibitory effect on protein translation (Ivanov et al., 2011; Sobala and Hutvagner, 2013; Krishna et al., 2019). Exogenous expression of 5’-tRNA halves but not 3’-tRNA halves have been found to induce phospho-eIF2α independent assembly of stress granules and inhibit protein translation in cultured U2OS cells (Emara et al., 2010; Ivanov et al., 2011). In particular, the 5’-Ala tRNA halves were found to bind with translation inhibitor YB-1, and cooperate with YB-1 to displace eIF4G/A from uncapped and capped mRNAs as well as dissociate eIF4F from the m^7^G cap, which leads to repression of protein translation (Ivanov et al., 2011).

Despite it being accepted that ANG induces tRNA halves *vivo* (Fu et al., 2009; Yamasaki et al., 2009; Su et al., 2019), there are many outstanding questions about the relationship between stress, ANG, enriched tRNA halves, and translational arrest that have yet to be answered. For instance, is ANG the only stress-induced enzyme responsible for tRNA halves? Is there any difference in the species of tRNA halves derived from different sources of stress? Is YB-1 indispensable for the translational inhibition by tRNA halves? A recent study showed that there are both ANG-dependent and ANG-independent tRNA halves induced by high concentration arsenite, which suggests that ANG may be not the only RNase responsible for generation of tRNA-halves under this particular stress condition (Su et al., 2019). Moreover, high concentrations of arsenite resulted in translational arrest in both wild-type and ANG depleted U2OS cells, suggesting that ANG regulated translational repression does not account for all of the translational control caused by stress (Su et al., 2019). To comprehensively answer these questions, more research needs to be done.

At cellular level, a variety of tRNA halves have been shown to exert divergent functions such as cell apoptosis, proliferation, and differentiation. ANG-induced tRNA halves were fund to interact with cytochrome c (Cyt c) to protect cells from chronic hyperosmotic stress-induced apoptosis (Saikia et al., 2014). Cyt c is a peripheral protein located at the mitochondrial inner membrane, where it functions to transport electrons between complex III and complex IV of the respiratory chain (Garrido et al., 2006). During apoptosis, the mitochondrial membrane is permeabilized, allowing Cyt c to be released into the cytoplasm (Reubold and Eschenburg, 2012). Cytosolic Cyt c binds apoptotic protease activating factor 1 (Apaf-1) (Zou et al., 1999) to activate caspase pathways, which leads to the morphological changes observed in apoptosis (Zou et al., 1999; Wang, 2001). A recent study detected 20 enriched tRNA halves in the Cyt c ribonucleoprotein complex, and showed that ANG treatment mitigated stress-induced apoptosis in primary neurons (Saikia et al., 2014). As a result, it proposed that ANG-induced tRNA halves bind to Cyt c and prevent it from activating caspases and apoptosis (Saikia et al., 2014).

Sex hormone-dependent tRNA halves were found to be enriched in estrogen receptor-positive breast cancer and androgen receptor-positive prostate cancer, where they enhanced cell proliferation (Honda et al., 2015). In addition, stable tRNA halves were found to be in extracellular exosomes and transferred between breast cancer cell cells (Gambaro et al., 2019). Proliferative cancer cells are prone to migration, escaping the immune response to form a metastatic niche that undergoes angiogenesis (Osaki and Okada, 2019). Therefore, tRNA halves seemingly participate in both intracellular and extracellular signal transduction in cancer.

Besides their role in cancer, tRNA halves also define the cellular state of mESCs (Krishna et al., 2019). Sequencing of small RNAs in mESCs under several differentiation regimens revealed that tsRNAs such as 5’-Gln-, Glu-, Val-, and Gly-tRNA halves were enriched in differentiated cells compared with isogenic stem-like cells (Krishna et al., 2019). Transfection of mimics of these 5’-tRNA halves inhibited pluripotency, whereas blocking these 5’-tRNA halves using antisense oligonucleotides enhanced cell pluripotency (Krishna et al., 2019). This study also identified tsRNA associated proteins such as IGF2BP1, YBX1, and RPL10 by pulldown assays flowed with mass spectrometry, and showed that binding of 5’-tRNA halves with IGF2BP1 prevented it from binding to c*-myc* mRNA; thereby, facilitating differentiation of mESCs (Krishna et al., 2019).

### Transfer RNA Fragments

At molecular level, tRFs have been demonstrated to be involved in regulating mRNA stability (Haussecker et al., 2010; Kumar et al., 2014; Kuscu et al., 2018), translation (Kim et al., 2017), and retro-element regulation (Boskovic et al., 2020). The tRF-5s and tRF-3s were found to be associated with the human Argonaute proteins AGO1, 3, and 4 by photoactivatable ribonucleoside-enhanced crosslinking and immunoprecipitation (PAR-CLIP) in HEK293 cells (Kumar et al., 2014), which raised the question of whether tRFs are involved in gene silencing pathways like miRNAs. miRNAs usually harbor 7–8 nucleotide long seeding sequences at their 5’ end to base pair with the 3’UTR of mRNAs (Bartel, 2009) at the same time nucleotide positions 8–13 interact with AGO (Hafner et al., 2010; Kumar et al., 2014). Interestingly, tRF-5s and tRF-3s were found to be associated with AGO in a miRNA like pattern (*i.e.* tRF-3s interact with AGO at nucleotide positions 8–12 and tRF-5s binds to AGO at nucleotide position 7 (Kumar et al., 2014). Additionally, thousands of RNAs were found to interact with tRF-3s and tRF-5s *via* AGO1 by human AGO1 cross-linking, ligation, and sequencing of hybrids (CLASH) (Kumar et al., 2014).

A recent study revealed that tRF-3s regulate mRNA expression *via* AGO-dependent and Dicer-independent pathways (Kuscu et al., 2018). The tRF-3s were demonstrated to be associated with Argonaute by immunoprecipitation followed by northern blotting (Kuscu et al., 2018). Transfection of tRF-3s decreased luciferase activity of mRNAs containing the complementary sequence of tRF-3s (Kuscu et al., 2018). This regulation of luciferase activity by tRF-3s was abolished by depletion of Argonaute proteins but not Dicer (Kuscu et al., 2018). In addition, the tRF-3s were also found to be associated with GW182/TNRC6 proteins (Kuscu et al., 2018), which are critical players in assisting mRNA degradation processes with RNA-induced silencing complexes (RISCs) (Eulalio et al., 2009). Collectively, these findings illustrated the mechanism by which tRFs base-pair match with target RNAs, and slice RNAs by associating with Argonaute and GW182/TNRC6 proteins (Eulalio et al., 2009). Not only have tRFs been found to be loaded on Argonaute proteins, but also the loading itself is cell-type-specific (Telonis et al., 2015b).

Apart from regulation of mRNA degradation, tRF-3s were also determined to be able to influence proteomics by affecting ribosomal biogenesis (Kim et al., 2017). The tRF-3s from LeuCAG tRNAs were found to bind to ribosomal protein mRNAs *RPS28* and *RPS15* by base-pairing (Kim et al., 2017). Inhibition of LeuCAG tRF-3s resulted in reduced translation of *RPS28* and *RPS15* mRNAs, which decreased abundance of 40S ribosomal subunits and eventually led to cell apoptosis (Kim et al., 2017). Furthermore, tRFs have also been shown to be associated with RNA binding proteins to affect gene expression. The tRF-2s derived from tRNA-Glu in breast cancer cells were shown to harbor YBX1 binding motifs and able to bind YBX1 protein, thus displacing the 3’UTR of oncogenic transcripts from YBX1 and suppressing the stability of oncogenic transcripts (Goodarzi et al., 2015). Similarly, several tRFs from nuclear tRNA-His, tRNA-Ala, and mitochondrial tRNA-Glu were found to harbor HuR binding motifs in breast cancer datasets (Telonis and Rigoutsos, 2018). Since HuR is involved in multiple biological functions including alternative splicing (Zhu et al., 2006; Izquierdo, 2008; Zhou et al., 2011; Akaike et al., 2014), alternative polyadenylation (Zhu et al., 2007; Dai W. et al., 2012), stabilizing mRNA transcripts (Fan and Steitz, 1998; Peng et al., 1998; Wang et al., 2000a; Wang et al., 2000b; Sengupta et al., 2003), destabilizing transcripts (Kim et al., 2009; Chang et al., 2010; Cammas et al., 2014), and mediating translation efficiency (Kullmann et al., 2002; Gorospe, 2003; Mazan-Mamczarz et al., 2003; Glorian et al., 2011), it is conceivable that tRFs may interact with HuR similar to YBX1. The molecular functions of tRFs associated with RNA binding proteins such as HuR remain to be fully understood. Besides these effects on mRNA stability and translation, particular tRFs were found to modulate histone expression and mediate reverse transcriptional and post-transcriptional regulation of endogenous retro-elements (Schorn et al., 2017; Boskovic et al., 2020). The regulation of tRFs on retro-elements not only helped to protect genome integrity, but could also regulate the expression of protein-coding genes through these embedded retro-elements in their introns and/or exons (Sharma et al., 2016; Boskovic et al., 2020).

The multiple functions of tsRNAs in various pathways demonstrates their critical role in biological processes such as apoptosis, proliferation, and differentiation as well as (Lee et al., 2009; Hanada et al., 2013; Kim et al., 2017) in pathological conditions such as neurodegenerative diseases (Hanada et al., 2013) and cancer (Goodarzi et al., 2015).

## Transfer RNA-Derived Small RNAs in the Heart

Investigation of the expression and function of tsRNAs in the heart has just started, which opens up both opportunities and challenges. Previous studies have shown the existence of tsRNAs in the heart (**Table 2**). 5’ tRNA halves from Val- (Fu et al., 2009; Dhahbi et al., 2013) and Gly-tRNAs (Dhahbi, 2015) (Dhahbi et al., 2013) were detected in mouse hearts by northern blot analysis. The 5’ leader-exon tRFs from Tyr-tRNAs are also expressed in mouse hearts, and their levels were augmented upon CLP1 deletion (Hanada et al., 2013). CLP1 is a component of the mRNA 3’ end processing complex, and it has been found to be associated with the TSEN complex and, potentially, contributes to pre-tRNA splicing (Hanada et al., 2013). Depletion of CLP1 led to accumulation of Tyr-5’ leader-exon tRFs in multiple tissues including the cortex, spinal cord, heart, and kidney, and eventually results in progressive motor neuron loss (Hanada et al., 2013). Transgenic expression of CLP1 in motor neurons can rescue impaired neuronal function in CLIP1 knockout mice (Hanada et al., 2013), but it remains elusive how these Tyr- 5’ leader-exon tRFs in cardiac tissue may affect heart function. Specific tRF-3s and tRF-5s were also detected human heart tissues. For example, tRF-3s from Arg- and Gln-tRNA, as well as tRF-5s from Gly- and Cys- tRNAs were detected in human heart tissues (Torres et al., 2019).

**Table 2:** Summary of transfer RNA-derived small RNAs (tsRNAs) expressed in cardiac tissue.

tsRNA types	Examples of tsRNA	References
5’ tRNA halves	5’-Val-tRNA halves	(Fu et al., 2009; Dhahbi et al., 2013)
5’ tRNA halves	5’-Gly-tRNA halves	(Dhahbi et al., 2013; Dhahbi, 2015)
5’ leader-exon tRFs	tRF-Tyr	(Hanada et al., 2013)
tRF-3s	tRF-Arg; tRF-Gln	(Torres et al., 2019)
tRF-5s	tRF-Gly; tRF-Cys	(Torres et al., 2019)
tRF-5s	tRF-Gly	(Shen L. et al., 2018)

## Role of Transfer RNA-Derived Small RNAs in Cardiac Hypertrophy

A very recent study identified tRF-5s enriched in isoproterenol (ISO)-induced hypertrophic rat hearts by small RNA transcriptome sequencing, and indicated that these tRF-5s may contribute to intergenerational inheritance of cardiac hypertrophy (Shen L. et al., 2018). These tRF-5s were demonstrated to bind to the 3’UTR of the hypertrophic regulator *Timp3* mRNA to inhibit its expression, leading to hypertrophy of cardiomyocytes (Shen L. et al., 2018). Importantly, these tRFs were found enriched in sperm from ISO-induced hypertrophic mice compared to healthy male mice (Shen L. et al., 2018). In addition, the F1 offspring derived from ISO-treated mice exhibited increased cardiac muscle fiber breakage, hypertrophic marker gene expression, cell apoptosis, and fibrosis in their hearts when compared to the F1 from healthy controls (Shen L. et al., 2018). Therefore, the study raised a very intriguing question of whether tsRNAs induced by cardiac hypertrophy could be inherited by the next generation and lead to pathogenesis. In fact, there are several lines of evidence consistently indicating that tsRNAs are enriched in sperm (Chen et al., 2016; Sharma et al., 2016; Natt et al., 2019; Sarker et al., 2019; Zhang et al., 2019). Some studies demonstrated the intergenerational inheritance of tsRNAs by injecting tsRNAs from the sperm of males fed a high fat diet into normal zygotes, and showed the offspring had altered expression of metabolic pathway components in addition to developing a metabolic disorder (Chen et al., 2016; Sarker et al., 2019). Therefore, it would be interesting to investigate more thoroughly whether tsRNAs-induced cardiac hypertrophy could also be inherited, which may identify novel therapeutic targets.

At the molecular level, the tRF-5s may not be the only functional tsRNAs involved in cardiac hypertrophy. Deep small RNA sequencing with advanced bioinformatic tools could help to identify or verify extensive tsRNAs in cardiac hypertrophy. High-throughput sequencing combined with immunoprecipitation (*i.e.* RNA immunoprecipitation (RIP)-seq) could be employed to detect Argonaute protein associated tsRNAs involved in cardiac hypertrophy. It would be also important to test whether neutralization of tsRNAs by antisense oligonucleotides could rescue the heart from fibrosis and the hypertrophic response. Moreover, because tRF-5s can inhibit retro-element transcription and regulate Cajal body biogenesis (Boskovic et al., 2020), it would be interesting to test whether these functions are all or partly associated with tRF-5-mediated cardiac hypertrophy. Furthermore, upon having defined specific tsRNAs involved in cardiac hypertrophy, the mRNA networks which are associated with tsRNAs in cardiac hypertrophy could be explored by pulling down mRNAs in hypertrophic hearts with *in vitro* transcribed tsRNAs that are labeled with digoxigenin or biotin. Alternatively, CLASH-seq experiments could be employed to directly crosslink endogenous tsRNA-mRNA hybrids in hypertrophic hearts for detection. On the other hand, tsRNA-associated protein networks could be determined through tsRNA pull down assays followed by mass-spectrometry or western blotting. It is anticipated that identification of cardiac hypertrophy associated tsRNAs and further defining their function in the heart will shed light onto novel therapeutic targets and approaches to treat cardiac disease.

## Role of Transfer RNA-Derived Small RNA Inducers and Regulators in Cardiac Hypertrophy

While increasing evidence supports the direct involvement of tsRNAs in the heart, we may also learn how tsRNA biogenesis-related “inducers” and “regulators” participate in governing cardiac function and hypertrophy. The inducers and regulators mentioned here refer to currently known factors that control expression of tsRNAs.

### Oxidative Stress in Cardiac Hypertrophy

Cardiac cells undergo pathological hypertrophy in response to mechanical stress. Although it is an adaptive process to increase contractility (*i.e.* compensated hypertrophy), it eventually leads to a high risk for heart failure through pathological remodeling (*i.e.* decompensated hypertrophy) (Frey and Olson, 2003; Diwan and Dorn, 2007; Nakamura and Sadoshima, 2018). Oxidative stress is an important inducer of this response (Takimoto and Kass, 2007; Maulik and Kumar, 2012). It occurs when excessive reactive oxygen species (ROS) are produced that cannot be offset by the intrinsic antioxidant defenses (Takimoto and Kass, 2007). ROS include superoxide and hydroxyl radicals as well as hydrogen peroxide (Takimoto and Kass, 2007). Because these molecules are inducers of tRNA halves (Thompson et al., 2008; Yamasaki et al., 2009) it would be interesting to study their role in oxidative stress-induced cardiac hypertrophy. Specifically, ROS induces mitochondrial DNA mutations, damages mitochondrial membrane permeability, as well as the respiratory chain and anti-oxidant defenses (Guo et al., 2013), which can further trigger cell apoptosis through mitochondrial stress and downstream signaling pathways (Chen et al., 2018). Mitochondrial dynamics and metabolism have been found to play a pivotal role in regulating differentiation of stem cells to cardiomyocytes (Chung et al., 2007; Porter et al., 2011); maintaining cardiomyocyte function (Piquereau et al., 2013; Eisner et al., 2017; Zhao et al., 2019), and mediating hypertrophy of cardiomyocytes (Rosca et al., 2013; Pennanen et al., 2014). The intrinsic links between ROS, mitochondria biology, and cardiac hypertrophy/cardiac function makes it an intriguing area to explore how tsRNAs might be functionally involved in any of these processes. Although not much research has been done, there are several lines of evidence indicating the existence of mitochondrial-derived tsRNAs in humans (Telonis et al., 2015b; Natt et al., 2019). Moreover, mitochondrial-derived tsRNAs were found to be enriched in sperms from people eating a high-sugar diet for a week compared to sperms from the same people eating a normal diet (Natt et al., 2019). These findings imply a potentially significant role for mitochondrial tsRNAs in intergenerational inheritance. As mentioned above, cardiac hypertrophy has been shown to affect offspring through sperm tsRNAs, it would therefore be extremely interesting to unveil the potential role of mitochondrial tsRNAs in cardiac function, and decipher whether these small ncRNAs could lead to intergenerational inheritance of cardiac hypertrophy. On the other hand, a very recent study showed that a paternal low-protein diet promoted ROS production in the testicular germ cells, which led to ATF7 activation and further reduced H3K9me2 expression (Abel and Doenst, 2011; Zhang and Chen, 2020). The altered epigenetic status affected tsRNA biogenesis and their expression profile in the spermatozoa, which resulted in intergenerational effects (Abel and Doenst, 2011; Zhang and Chen, 2020). This not only reinforced the tsRNA function in intergenerational inheritance but also revealed the link between oxidative stress, tsRNA generation, and epigenetic regulation. These studies also raised questions about whether oxidative stress-induced cardiac hypertrophy could transmit intergenerationally, and if so, whether ATF7 and/or epigenetic alterations could be considered as therapeutic targets for inherited cardiac hypertrophy.

### Aging and Caloric Intake in Cardiac Hypertrophy

Aging and excessive caloric intake are highly associated with cardiac hypertrophy (Dong et al., 2007; Dai D. F. et al., 2012; Chiao and Rabinovitch, 2015; Wang et al., 2015). Elevated ROS released by mitochondria has been proposed to be the primary driving force of aging and a major determinant of lifespan (Harman, 1972; Miquel et al., 1980; Dai et al., 2014). Consistent with this, ROS production by mitochondria, as well as disrupted mitochondrial function, have been shown in the aging brain, heart, and skeletal muscle tissues (Sawada and Carlson, 1987; Capel et al., 2005; Retta et al., 2012; Tocchi et al., 2015; Lesnefsky et al., 2016; Boengler et al., 2017; Grimm and Eckert, 2017). Aging intertwines with ROS related mitochondrial DNA mutation, respiratory chain deterioration, and mitochondrial metabolism impairment (Fleming et al., 1982; Wallace, 1992). The disrupted mitochondrial function along with aging increases production of ROS, which, in turn (as introduced above), could affect mitochondria by damaging mitochondrial DNA and causing functional deterioration, which is referred to as the “vicious cycle” concept (Alexeyev et al., 2004; Dai et al., 2014). Therefore, age-related cardiac hypertrophy is a complex syndrome from a mitochondrial function and oxidative stress perspective. On the other hand, 3’-tRFs and 5’-tRFs were detected in rat brain, and the 3’-tRFs were found to be increased with rat age (Karaiskos and Grigoriev, 2016). Thus it is conceivable that tsRNAs might be enriched in aging hearts, and age-related hypertrophic hearts. Moreover, there is also an interesting link between calorie restriction and aging retardation as well as cardiac functional improvement. A number of studies suggest that caloric restriction can prevent or reduce cardiac hypertrophy, improve cardiac function, and even retard aging (Yu, 1994; Cruzen and Colman, 2009; Dolinsky et al., 2010; de Lucia et al., 2018; An et al., 2020). Although the mechanisms are still unclear, there is evidence showed that aging and caloric restriction can modulate specific 5’ tRNA halves (Dhahbi et al., 2013). Val- and Gly-5’-tRNA halves were found to be enriched in aged mouse serum when compared to young mice and that caloric restriction mitigated these differences (Dhahbi et al., 2013). In addition, as introduced above, several studies have shown that high sugar or high fat diets affect mitochondrial and other tsRNA expression profiles in sperm (Chen et al., 2016; Natt et al., 2019; Sarker et al., 2019). So, it would be interesting to determine the role of tsRNAs in the dynamics of aging, oxidative stress, and metabolism during development of cardiac hypertrophy.

### ANG in Cardiac Hypertrophy and Heart Failure

ANG is a major inducer of tRNA halves (Thompson et al., 2008; Fu et al., 2009; Yamasaki et al., 2009; Su et al., 2019), and several studies suggest ANG is involved in cardiac hypertrophy and heart failure (Patel et al., 2008; Jiang et al., 2014; Yu et al., 2018; Oldfield et al., 2020). ANG not only functions as an RNase, but is also a potent stimulus for angiogenesis (Tello-Montoliu et al., 2006). Pro-angiogenic factors such as vascular endothelial growth factor (VEGF), basic fibroblast growth factor, and ANG, are involved in the development of cardiac hypertrophy (Oldfield et al., 2020). Cardiomyocytes secret pro-angiogenic molecules to support vascular growth to increase blood flow in the hypertrophic heart (Oldfield et al., 2020). Interestingly, ANG has been proposed to be a biomarker for left ventricular systolic dysfunction and heart failure (Patel et al., 2009; Jiang et al., 2014; Yu et al., 2018). A clinical study collected serum from 16 patients with heart failure with preserved ejection fraction (HFpEF) and 16 healthy individuals, and found that ANG differed the most among 507 proteins between the two groups (Jiang et al., 2014). Particularly, the average serum ANG level was 374 ng/ml (%95 CI 348–400 ng/ml) in healthy controls and 477 ng/ml (95% CI 438–515 ng/ml) in HFpEF patients (P<0.001) (Jiang et al., 2014). A follow-up study were performed in 203 patients with coronary heart failure (CHF), 413 coronary heart disease patients without chronic heart failure (also called CHD disease controls), and 53 healthy controls to explore the potential utility of ANG as a biomarker (Yu et al., 2018). The CHF group were further subgrouped into heart failure with reduced ejection fraction (HFrEF) and heart failure with preserved ejection fraction (HFpEF). It was found that the CHF group had higher ANG plasma levels compared with either healthy controls or CHD disease controls. The HFrEF patients had higher ANG plasma levels compared with HFpEF patients or CHD disease control patients.

Besides cardiac hypertrophy and heart failure, ANG has been linked to other diseases such as diabetes (Altara et al., 2018) and hypertension (Marek-Trzonkowska et al., 2015). Therefore, it is likely that dysregulation of ANG in cardiac hypertrophy, heart failure, and other cardiovascular diseases may lead to tsRNA dysregulation in the heart, and it is worth further investigating the potential biological function of ANG-induced tsRNAs in these instances.

### ELAC2 in Cardiac Hypertrophy

Cytosolic ELAC2 contributes to the generation of tRF-1 (Lee et al., 2009), while mitochondrial ELAC2 is responsible for mt-tRNA processing (Brzezniak et al., 2011; Siira et al., 2018). A few studies suggest that ELAC2 is implicated in mitochondrial disorders and cardiac hypertrophy (Haack et al., 2013; Siira et al., 2018). Cardiac-specific ELAC2 deletion in mice leads to reduced mitochondrial protein synthesis, OXPHOS biogenesis, mitochondrial oxygen consumption, and disruption of regulatory non-coding RNAs (Siira et al., 2018). The combined disruptive effects causes early-onset dilated cardiomyopathy and premature death by 4 weeks (Siira et al., 2018). Furthermore, mutations in the human *ELAC2* gene is associated with mt-tRNA processing defects associated with cardiac hypertrophy (Haack et al., 2013). Unfortunately, the underling mechanisms remain unclear and the role of mitochondria in cardiac hypertrophy and heart failure is dynamic and complicated.

During the development of cardiac hypertrophy, mitochondria compensate by increasing oxidative phosphorylation and ATP synthesis (Rabinowitz and Zak, 1975); however, this can result in mitochondrial dysfunction (Zhou and Tian, 2018). This complication can disrupt the electron transport chain and APT production, as well as affecting the modification of proteins, calcium homeostasis, and inflammation, which are important contributors to cardiac hypertrophy and heart failure (Abel and Doenst, 2011; Rosca et al., 2013; Zhou and Tian, 2018). Consequently, it would be interesting to determine the following: 1) ELAC2 function in mitochondria during compensation and decompensation, 2) ELAC2 levels in hypertrophic hearts, and 3) the functional role of these tsRNAs in cardiac hypertrophy.

### Hypoxia in Cardiac Hypertrophy

Though hypoxia can generate tRNA halves (Fu et al., 2009), it is also associated with cardiac hypertrophy due to increases in oxygen consumption and reductions in the blood supply to the enlarged heart (Kumar et al., 2018). Sustained hypoxia leads to reprogramming of gene expression and metabolism, which further aggravate decompensated cardiac hypertrophy and, ultimately, lead to heart failure (Giordano, 2005; Chu et al., 2012; Mirtschink and Krek, 2016). So, it is not surprising that an ischemic injury causes up-regulation of Val-and Gly-tRNA derived tRF-5s in the rat brain as determined using deep sequencing (Li et al., 2016). Consistent with this observation, these tRF-5s were also enriched in a hind limb ischemia model and in hypoxic endothelial cells (Li et al., 2016). Moreover, exogenously expressed Val- and Gly-tRF-5s repress cell proliferation, migration, and tube formation in hypoxic endothelial cells (Li et al., 2016). Coincidently, tsRNAs from Val- (Fu et al., 2009) (Dhahbi et al., 2013) and Gly-tRNAs (Dhahbi, 2015) (Dhahbi et al., 2013) were documented to be detectable in mouse hearts by northern blot. Therefore, it would be worthwhile to test whether these tsRNAs are regulated by hypoxia during cardiac hypertrophy.

## Conclusion and Perspectives of Transfer RNA-Derived Small RNAs in Cardiovascular Biology and Medicine

The tsRNAs are newly-identified sncRNAs derived from endonucleolytic cleavage of pre-tRNAs or mature tRNAs. Based on differences in cleavage sites and the size of cleavage products, tsRNAs are divided into tRNA halves and tRFs. tRNA halves can regulate stress granule assembly and protein translation (Emara et al., 2010; Ivanov et al., 2011), and affect cell apoptosis (Saikia et al., 2014), proliferation (Honda et al., 2015) and differentiation (Krishna et al., 2019). tRFs are also involved in mRNA stability regulation (Haussecker et al., 2010; Kumar et al., 2014; Kuscu et al., 2018), translation regulation (Kim et al., 2017), and retro-element transcriptional regulation (Boskovic et al., 2020). These tsRNAs play important roles in physiological and pathological conditions such as development (Krishna et al., 2019), aging (Dhahbi et al., 2013), neurogenerative diseases (Hanada et al., 2013), cancer (Honda et al., 2015), and cardiovascular diseases (Shen L. et al., 2018).

As tsRNAs are relatively new, limited studies have been performed on their role in cardiac function. However, several studies suggest that tsRNAs exist in cardiac tissues (Fu et al., 2009; Dhahbi et al., 2013; Dhahbi, 2015). There is also a study that shows tsRNAs are implicated in the inheritance of cardiac hypertrophy (Shen L. et al., 2018). Furthermore, there appears to be a relationship between cardiac hypertrophy and tsRNA inducers or regulators such as oxidative stress (Takimoto and Kass, 2007; Maulik and Kumar, 2012), hypoxia (Kumar et al., 2018) (Giordano, 2005; Chu et al., 2012; Mirtschink and Krek, 2016), ANG (Patel et al., 2009; Jiang et al., 2014; Yu et al., 2018), ELAC2 (Haack et al., 2013; Siira et al., 2018), aging, and caloric intake, (Dong et al., 2007; Dai D. F. et al., 2012; Chiao and Rabinovitch, 2015; Wang et al., 2015), which indicates an important role for tsRNAs in cardiac hypertrophy.

Therefore, there are a number of research opportunities to examine the role of tsRNAs in cardiac hypertrophy and other cardiac diseases. While it is not clear how global tsRNAs are changed during the development of cardiac hypertrophy, although tRF-5s were identified in ISO-induced hypertrophy (Shen L. et al., 2018). Whether other inducers modulate tsRNA in different cardiac hypertrophy models remains unknown. If this happens to be the case, it would be interesting to see if different hypertrophy inducers generate different tsRNAs and understand their biological function. As we discussed, cardiac hypertrophy can be categorized into compensation and decompensation stages (Frey and Olson, 2003; Diwan and Dorn, 2007; Nakamura and Sadoshima, 2018). Dissecting the role of tsRNAs in these stages may provide new perspectives or therapeutic targets. Since there is very limited research that has investigated the role mitochondrial tsRNAs, most of the molecular and biological functions of tsRNAs described here are cytoplasmic tsRNAs generated from nuclear-encoded tRNAs. So, mitochondrial-encoded tRNAs (Suzuki et al., 2011) and those that can shuttle between the cytoplasm and mitochondria (Rubio et al., 2008; Mercer et al., 2011) represent an opportunity for further investigation.

**Figure 3:**
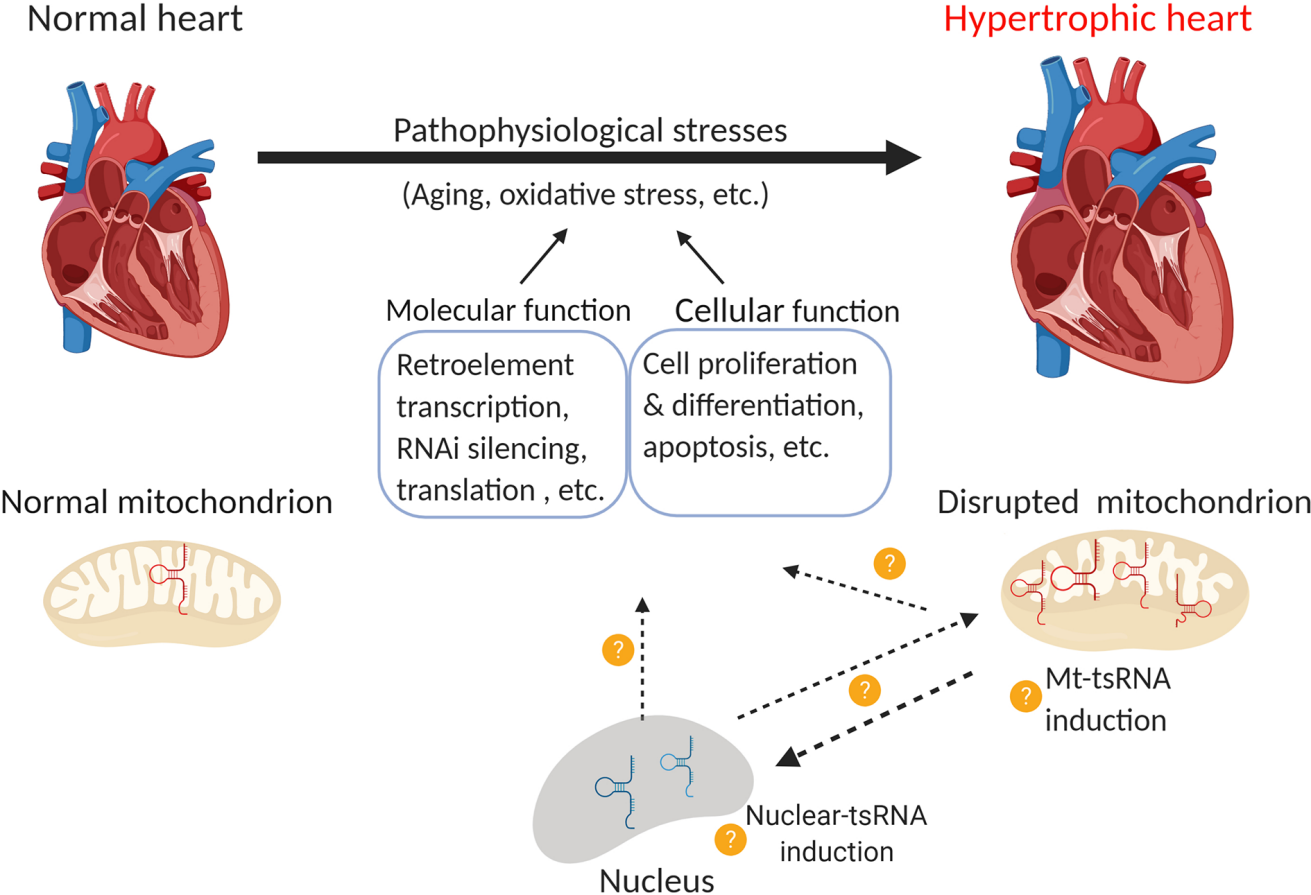
Function of nuclear and mitochondrial tsRNAs in cardiac hypertrophy. Pathological stress (*e.g.* oxidative stress) leads to the development of cardiac hypertrophy. Emerging evidence indicates that the disruption of mitochondrial tsRNAs plays a role in this process. Consequently, it would be interesting to investigate the following questions: 1) Whether nuclear-tsRNAs or mt-tsRNAs are induced or dysregulated during cardiac hypertrophy, 2) Whether any of these tsRNAs exert molecular functions such as the regulation of retro-element transcription, RNAi silencing, and translation, or cellular functions such as cell proliferation, differentiation, and apoptosis, and 3) Whether nuclear-tsRNAs and mt-tsRNAs shuttle between the nucleus and mitochondria, and if their function in different organelles affects cardiac hypertrophy (Created with BioRender.com).

Several lines of evidence demonstrate that mitochondrial tsRNAs differ from nuclear tsRNAs in terms of their sequence and size (Hirose et al., 2015; Telonis et al., 2015b; Loher et al., 2017). Mitochondrial tsRNAs may also contribute to miRNA biogenesis in these organelles (Venkatesh et al., 2017). As mitochondria are heavily involved in hypertrophic responses (Ballinger, 2005; Abel and Doenst, 2011; Rosca et al., 2013) and mt-tRNA mutations are associated with cardiovascular disease (Jia, Wang et al., 2013; Scheibye-Knudsen et al., 2015), there is a pressing need to uncover the role of tsRNAs in the heart. Lastly, tRNAs (especially mt-tRNAs) undergo extensive post-transcriptional regulation that may affect their function (Suzuki and Suzuki, 2014; Lyons et al., 2018; Richter et al., 2018). For instance, cytosin-C5 tRNA methylation by DNMT2 and NSUN2 promoted tRNA stability (Tuorto et al., 2012). Deletion of DNMT2 caused upregulation of tsRNA-Gly in mouse sperm (Zhang et al., 2018), whereas loss of NSUN2 promoted tsRNA generation in tumors (Blanco et al., 2016). PUS7 is a pseudouridylation epigenetic “writer” of tRNAs, the deletion of which leads to altered expression of tsRNAs in embryonic stem cells, which further impairs tRF-mediated translation regulation and results in defective germ layer specification (Guzzi et al., 2018). Understanding these modifications may provide insights into tsRNA biology and their role in cardiac disorders and diseases (**Figure 3**).

tsRNAs have also been linked to the gene translational program in embryonic stem cells (Blanco et al., 2016; Krishna et al., 2019), thus it would be interesting to define the role of tsRNAs in cardiac development as well as differentiation of stem cells into mature cardiomyocytes. In addition, retro-elements are highly expressed in stem cells (Boroviak et al., 2018), whose regulation helps to determine cell differentiation and development (Schoorlemmer et al., 2014; Robbez-Masson and Rowe, 2015). As introduced above, tsRNAs are implicated in the regulation of retro-element expression (Schorn et al., 2017; Boskovic et al., 2020); therefore, it would be interesting to decipher whether tsRNAs are implicated in determining these developmental stages by regulating retro-element expression.

## Author Contributions

JC and D-ZW conceived the idea for this review. JC, D-ZW, and DC wrote and edited the review.

## Funding

Research in the Wang laboratory is supported by the NIH (HL138757, HL125925, and HL093242). JC is supported by an American Heart Association Postdoctoral Fellowship (18POST33990181).

## Conflict of Interest

The authors declare that the research was conducted in the absence of any commercial or financial relationships that could be construed as a potential conflict of interest.
